# Hospital and Institutional News

**Published:** 1912-04-20

**Authors:** 


### HOSPITAL AND INSTITUTIONAL NEWS.

### THE SINKING OF THE "TITANIC."

The terrible disaster to the White Star liner *Titanic*, which sank in the early hours of Monday morning, has involved both Europe and America in a common sorrow. She left Queenstown with nearly 2,200 persons, including the crew, on board, and as we go to press it is virtually certain that only 868 have been saved. Few, indeed, are the professions and interests which have not suffered loss, and we chronicle the names of four doctors who have not yet been accounted for. The first two of these are Mr. W. F. N. O'Loughton, ship's surgeon on the *Titanic*, though this name appears to be a mis-spelling of the O'Loughlin, with the same initials, qualifications, and appointments, mentioned in the Medical Directory; a second ship's surgeon was also on board, Dr. J. E. Simpson, M.B., of Belfast, who acted as assistant doctor. Among the passengers unaccounted for at the time of writing are the names of Dr. Arthur Jackson Brewe and Dr. W. E. Minahan, who presumably belong to the profession in the United States. These were both first-class passengers. As, however, the number of notified survivors is larger than the list of names at present come to hand, it is possible that this serious outlook may be modified. Nothing, however, save in the case of a few privileged families and groups of friends can lessen the force of this disaster, which can be called unparalleled without any exaggeration. It is no part of ours to enter into the general questions involved in this catastrophe, but the medical profession which has to insist on so many precautions on land will await with interest the inquiry as to whether or not the number of lifeboats provided was adequate in any real sense to the number of persons on board. The lesson of the disaster is that no ship is really unsinkable, and that once granted, the importance of such measures as a proper complement of lifeboats becomes overwhelming.

### A GREAT INFIRMARY SUPERINTENDENT RETIRES.

Dr. Henry Russell Bencraft, medical superintendent of Shirley Warren Infirmary, Southampton, has sent in his resignation. His retirement comes at the end of a long period of work, for he has had control of the Shirley Warren institution from its establishment in 1892. Before that date the patients, some of whom have known Dr. Bencraft for almost twenty years, were housed in the workhouse in St. Mary Street. The staff and patients, with every member of whom he shook hands at parting, now number 400, and the magnitude of his task will be realised when it is understood that the whole work of organising the new infirmary was concentrated in his hands. Dr. Bencraft, who qualified in 1885, studied at St. George's Hospital, and, we may add, was well known on the cricket field, has been presented with a silver salver and cake dish inscribed "Presented to Dr. Russell Bencraft by the medical and nursing staff and other officials of Shirley Warren Infirmary as a mark of respect and esteem on the occasion of his retirement from the position of medical superintendent of the infirmary." It would be hard to exaggerate the influence which Dr. Bencraft exerted or the popularity in which he is held. His work has been of immense value, and his retirement is a very great loss to Poor Law infirmary work in this country.

### PROPOSED "CHRISTIAN" HOSPITAL FOR LONDON.

Dr. L. G. Broughton, an American minister who is about to take charge of Christ Church, Westminster Bridge Road, announces his intention of starting a "Christian" hospital. It should be recalled that Dr. Broughton introduced a hospital so described to Atlanta, Georgia, and from there the movement has spread. "We shall," he says, "not only be a hospital for the very poor; we shall try to serve the man of small means who does not wish to depend on charity. We shall manage it as we do at Atlanta. The fees will be quite moderate, and a man may take a year or a year and a half to pay." It is amazing, however, to assume that the Christian hospital is an innovation in this country, which is the home of the voluntary system, and where the religious needs of the patients have been looked after from the first. Apart from this really monstrous assumption, we much doubt whether the profession or the public would take kindly to any such proposals.

### INEFFICIENT CONTROL IN A POOR LAW INFIRMARY.

A case exciting some comment has occurred at the Booth Hall Infirmary, Manchester, in consequence of an inquest held concerning the death of a woman patient. It appeared from the evidence that as the result of a fall the woman fractured the base of her skull on February 22, and her medical attendant, Dr. Yeoman, exactly a month later recommended her removal to the infirmary. A sister stated that though the receiving card mentioned the fractured skull, she did not think it necessary to mention the fact to the infirmary medical officer, Dr. Ramsbottom. He diagnosed the case as one of delirium following upon the taking of alcohol, and being confident in the correctness of his diagnosis, he did not receive nor demand a history of the case. He gave the cause of death in the certificate as peripheral neuritis and œdema of the lungs. Another practitioner who gave evidence stated that a month after the accident there would probably be no evidence of the fractured skull. The coroner remarked that everybody except the doctor who attended the patient up to her death knew about the fractured skull, but he did not say that Dr. Ramsbottom's failure to inquire into the history of the case was neglectful. The foreman of the jury, in giving the verdict of accidental death, expressed the opinion that there had been a great amount of neglect at the Booth Hall Infirmary in not reporting the case more fully. In our opinion neglect is not the word to use, for neglect implies a wilful disregard of humanity or duty. The moral of the case for all medical superintendents is that faulty administration and lack of proper control and discipline in the staff will lead to results which will be regarded as neglect by juries and laymen.

### CHARING CROSS HOSPITAL AND THE INSURANCE ACT.

Added significance was given to the annual meeting of the Hospital Saturday Fund from the fact that the speakers took up the defence of the voluntary system and expressed in strong terms their fears of the Insurance Act. After the report of the Fund had been adopted on the motion of Sir Savile Crossley, who emphasised the value of the voluntary system, Mr. Walter Alvey, Secretary of Charing Cross Hospital, moved a resolution urging "that there should be a provision under the National Insurance Act for payment to be made to hospitals for work done on behalf of insured persons, in such a way that the voluntary system is not interfered with." He calculated that 55 per cent. of the patients treated at Charing Cross Hospital would be insured.

### MR. WALTER ALVEY'S VIEW.

In the course of a lengthy address Mr. Alvey proceeded to ask whether these insured patients were to be taken away from the voluntary hospitals and treated elsewhere. Though opinions differed on the point, he thought that a large number would be taken away. If the effect of the working of the Act were to reduce the work of the voluntary hospitals, no one, he considered, would be more delighted than the authorities of the voluntary hospitals themselves, because at the present time the voluntary hospitals were sadly overburdened. In their out-patient departments the work was so heavy that it was impossible to give that amount of individual care which each case required. In the in-patient departments all the hospitals had heavy waiting lists. Some applicants waited for weeks, some for months, and some never got in at all. He often wondered what became of these people. The resolution was seconded by Mr. Thomas Bevan, and carried.

### DUBLIN HOSPITALS AND THE INSURANCE ACT.

An important deputation representing the Dublin hospitals has waited on the Insurance Commissioners, and through the chairman of the Joint Hospitals Committee, who submitted a written statement, has suggested that the only fair way of helping the hospitals would be by a capitation grant from approved societies and insurance committees, to be distributed among the hospitals on the work done. The chairman stated that the demands upon the hospitals were likely to increase from the fact that medical benefits were not given in Ireland, while their expenses would be increased in connection with their own staffs. Sir John Moore, M.D., feared that serious injury would result to clinical work, and the Medical School of Dublin would be imperilled if the hospital authorities had to close down wards or beds. Dr. Jellet then argued that the immediate effect of the maternity benefit, if it were worked according to the letter of the Act, would be to penalise every woman who enters a hospital for her confinement, and thereby to destroy the system which had made Dublin regarded as possessing the most famous teaching school for medical students and midwives in the Empire. Dr. Jellet urged that under section 12 (2) (*a*) the poor should be given clearly to understand that if individually they lost their maternity benefit by entering a hospital the money should be paid to their dependants, and that women attended by students and pupil nurses in the extern departments under the supervision of the hospital staff should be regarded for the purposes of the Act as attended by qualified medical men, and should be eligible for maternity benefits. The maternity hospitals should be given a capitation fee for each insured patient admitted to the gynæcological wards. Mr. Adam Lloyd Blood, a hospital governor, suggested that nurses should be treated as inmates, as mentioned in section 51, and should be declared exempt from liability to insure, in relief of the hospitals as employers; this to apply to probationers also.

### AN INSURANCE COMMISSIONER ON THE HOSPITALS' POSITION.

In reply the chairman of the Irish Commissioners, Mr. Glynn, suggested that the hospitals should bring a scheme as to how their position was to be relieved, but he thought that they had completely misunderstood the powers of the Irish Commissioners. We, he said, have no power to grant any sum to a hospital, nor any power to compel approved societies or insurance committees to do so. The only sections dealing with the question were 12 and 21. All agreements, he continued, with the hospitals must be made by the societies and insurance committees, and these bodies were not compelled either to contribute to the hospitals or to enter into agreements with the hospitals for the maintenance of their members therein. The elimination of medical benefits in the case of Ireland disposed of the contentions of those subscribers who threatened to withdraw their support on the ground that they were being taxed for having their sick dependants treated. As regards insuring hospital staffs the chairman said that hospitals should consider section 51, under which it was possible to claim exemption, though as a matter of fact a decision would soon be given as to liability of nurses and probationers to be insured. The points raised by Dr. Jellet would be discussed at a later meeting. This discussion has been worth quoting at some length because, though nothing can be conclusive till tested by the practical results of the Act, it is another proof of the wisdom of the Irish hospitals in making use of the ever-shortening interval between now and July 15 for raising their claims and affirming their position. In this respect they set an example which institutions over here would do well to follow.

### THE SCOTTISH MEDICAL INSURANCE COUNCIL.

To the six cardinal principles demanded by the British Medical Association as the condition of the acceptance by the medical profession of appointments under the Insurance Act, the Scottish Medical Insurance Council has added a seventh. At the first meeting of this body, held last Friday and Saturday in the Hall of the Royal College of Surgeons of Edinburgh, it was agreed that in addition to the famous six points as originally laid down, there should be required another, namely: "Disciplinary powers to be vested in some properly constituted medical body." The Council further resolved to inform the Scottish Commissioners at once of its existence and of its representative constitution, and to inform them also of its strong disapproval of the action of the Scottish Commissioners in appointing medical members of the Advisory Committee without reference to the wishes of the profession. Other resolutions were adopted, the general tone of which shows that the Scottish medical men are keeping a "stiff upper lip" over the Insurance Act; and it will not be surprising if the resistance of Scotland to the working of the Act is more determined even than that which is anticipated elsewhere in the United Kingdom. As to the seventh "point" which has thus been added to the original six, the feeling of the profession will doubtless be that it is admirable as it stands; the difficulty we foresee is that of getting the general public to accept it as a fair and reasonable demand, owing to the suspicion which is (unjustly) entertained of medical discipline.

### A MENTAL CASE IN AN INEBRIATES' HOME.

A patient's escape and death after a struggle in Lady Henry Somerset's Inebriates' Home at Duxhurst has been revealed in an inquest at Redhill. After previous inquiries as to a vacancy a telegram was received on Tuesday last week announcing the arrival of a lady patient. On her arrival with her nurse and brother it was found that the patient had previously drunk whisky and a medicated wine to excess, but that at the present time she was suffering from delusions, and that a few days before she had jumped out of a window and escaped to a cottage, where she remained till her removal to Duxhurst. The brother was then told that mental cases were not accepted, and that the patient was not considered a suitable case. He, however, begged Lady Henry Somerset to take the patient in, as she had travelled from Huntingdon that day and there was nowhere else to send her. Under pressure, therefore, the case was taken in temporarily. A mental nurse was telegraphed for and the patient was placed in a room on the ground floor. All was quiet till after midnight, when the patient left her bed, took possession of the key, which she prevented the nurse from regaining, and half an hour later began to struggle so violently that the nurse was overpowered. The patient then disappeared through the window into the grounds, and on the nurse's cries attracting attention a search was made, which next morning ended in the discovery of the patient dead in a field some quarter of a mile off. Dr. A. R. Walters, the superintendent of the Home, attributed death to syncope, and said it would have been inhuman not to receive the patient considering the late time of her arrival and her condition. In returning a verdict of Death from natural causes the jury added that they thought the staff had done everything possible. It will be noted that the patient was virtually repeating a previous escapade, but that the Home took the wise precaution of sending for a nurse specially trained in mental work. No blame, therefore, can besmirch this instance of humanity, except that for want of a bell the nurse had to scream to attract attention. But the case will be remembered as a tragic instance of the responsibilities and risks incurred by Homes in receiving, even from the highest motives, patients for whom they are not designed.

### DISPENSARY CHANGES AT SALFORD.

Mr. Robert Stonex, Honorary Secretary of the Greengate Dispensary, Salford, has resigned his post. He has held the appointment for ten years, and his successor is Mr. Percy Stowell. The new Secretary takes up the work at an important development of the dispensary's history. Not only has it completed almost half a century of work, but an extension is about to take place. In this connection it is interesting to remember that in 1904 an in-patients' department of nine beds was opened, chiefly for paralytic cases.

### NEW COTTAGE HOSPITAL SECRETARY.

Mr. P. Mallinson, Honorary Secretary of the Brentford Cottage Hospital and Dispensary, has resigned his work for the hospital, but has promised to continue his duties at the dispensary for at least a year. One of the problems which he wishes to settle in connection with the dispensary is the best way of finding out those who do not use their tickets. His successor as honorary secretary to the hospital is Mr. Bellamy. In some cases this and similar offices in cottage hospitals are carried on in the same family by different generations, usually to the great advantage of the institution, but where this is not the case it is sometimes difficult to find applicants for an honorary post which entails a very large and growing amount of ungrudging attention to detail.

### THE LATEST ACHIEVEMENT AT LEICESTER.

The Swithland Convalescent Home for Women, near Woodhouse Eaves, which has been built by the Leicester and County Hospital Saturday Fund, was opened last week by Mrs. C. J. Bond. The home has already been referred to, but we may just remind our readers that an impetus was given to its foundation by the bequest of Mr. E. Higgs, which realised £10,000. As Sir Edward Wood remarked, another important period of the Saturday Fund began with this opening. It was only eight years since the Fund had been established, and in its first year it raised about £4,000, and last year, as The Hospital has recorded, the total reached the astonishing sum of nearly £15,000. They had long hoped to have a women's convalescent home, ever since, in fact, the convalescent home for men had been opened at Desford Hall, seven years ago.

Some £700 has been subscribed towards the furnishing of the Home, but the total expense under this head is expected to be £1,200. The architects are Messrs. Seale and Riley. As we have remarked before, the Leicester Hospital Saturday Society sets an example to the whole country in the way in which year by year it consolidates the support of the Infirmary, which, through this Fund, has become the centre of a splendid network of enthusiasm which has found expression in many activities of which this elaborate Convalescent Home is only the latest example. We cannot praise too strongly either the Infirmary or the Saturday Fund, both of which represent at Leicester some of the best fruits that the voluntary system anywhere can show. Mr. Harry Woolley is the Secretary of the Saturday Society.

### STAFF DISPUTE AT WOOD GREEN CONTINUED.

We have referred already to the dispute which has been in progress at the Wood Green Hospital concerning the numbers of local medical men who should be allowed to visit their patients in the hospital. Originally, as we stated, the number was twelve. Then it was decided to raise it to eighteen, which has meant fifteen in practice, as three more are to be added, but at a later date. So far from this settling the matter, however, the first meeting of the newly-elected Council—at which, by the way, Mr. H. Smith took the place of the retiring chairman, Mr. Denham—once more discussed it. Mr. Searle, who represents the Southgate District Council, which subscribes £80 to the hospital, again took the matter up in a letter read by Mr. Barber, the secretary. In brief, the situation is that there are seventy or eighty local doctors; that the staff there having the right to visit their patients is, though raised, only fifteen; that these decline to add to their number, and that the Hospital Committee is afraid that if Southgate's request put forward by Mr. Searle for giving every practitioner the right to visit his patients be refused the Southgate subscription may be discontinued. In all cottage hospitals—the best prevailing rule is to allow all the local doctors the right of visiting their own patients. There seems no adequate reason why this should not be the rule at Wood Green, for the hospital cannot be considered large enough to make the overcrowding of doctors which is feared a matter of difficulty. Only a very small proportion of the local practitioners in a hospital of this size would have their own patients in the wards.

### A REMARKABLE CAREER.

The death of Dr. William Ogle at the ripe age of eighty-four removes a striking figure who in his time played many parts and influenced the development of public health, of medicine, and of institutional progress. He was the son of a Regius Professor of Medicine in Oxford University, and after a brilliant career at that seat of learning he took orders. He soon abandoned the Church, however, and studied medicine at St. George's Hospital. At the medical school of this institution he became lecturer on physiology, and it is said that his brilliant teaching of that (then) new science attracted hearers from outside the circle of medical students. Eventually he became assistant physician to the hospital in 1869, only to resign the post for reasons of health three years later. He next took up public health work, and was Medical Officer of Health for South Herts; but he "found himself" when in 1880 he became Superintendent of Statistics in the Registrar General's Office. From that time onwards he poured forth a sequence of reports and statistical inquiries which ranged over an enormous field, and he found time to serve on Royal Commissions and to give evidence before several Committees. Nor did these voluminous technical works represent the whole of his prodigious industry. Dr. Ogle was a classical scholar, and he published translations of Aristotle and other classical authors which of themselves would have made his reputation. His very numerous scientific writings between 1880 and 1903 cannot be enumerated here; but they bore the unmistakable imprint of hard work, of scholarly composition, and of a most uncommon personality. Dr. Ogle knew what he wanted to say, and said it regardless of consequences; but his conclusions were founded upon honest work conducted in a truly scientific spirit, and it was seldom that they were successfully challenged.

### AN AID TO THE CHARING CROSS APPEAL.

In the second year of the Charing Cross Hospital appeal the authorities are following the example of other institutions and arranging for a great social function. Lady Juliet Duff is organising a Café Chantant at the Savoy Hotel on May 15, for which the management have kindly lent the whole of the ground floor as well as their celebrated orchestra. The event has been already patronised extensively by prominent people, and promises to be one of the fashionable events of the season. We trust that the deserving hospital for which the affair is being arranged will benefit substantially in a pecuniary sense, and that attention once more will be attracted to the appeal.

### AN AUSTRALIAN DOCTOR IN THE PULPIT.

In this country we are not well accustomed to the idea of the medical man ascending the ecclesiastical pulpit, though the suggestion was recently made in the North that practitioners should relieve the clergy of their duty on Hospital Sunday in this respect, but Australia is less conservative. Thus Dr. John William Springthorpe, M.D., in-patient physician at the Melbourne Hospital, gave by request a Lenten address on tuberculosis in St. Paul's Cathedral of that city. Dr. Springthorpe himself is a Congregationalist. He graduated at Melbourne in 1879, obtained the M.R.C.P. of London in 1881, and three years later gained the Melbourne M.D. In the course of his address he remarked that nearly every suburb in Melbourne raised its voice against having sanatoria established within its borders. It would seem from this that the wide publicity now given to warnings against spitting has succeeded in creating an exaggerated fear of infection in the public mind. In hotels, of course, the consumptive is often treated as a leper, but we do not appear to be so morbidly afraid of his propinquity as the Melbourne suburbs. Indeed, the recent opposition to the building of a consumptive ward in an Irish town brought an appreciable amount of ridicule upon the protestors. There may be therefore some need in Melbourne for the doctor in the pulpit, not merely to warn men against the dangers of consumption and to emphasise reasonable precautions, but to teach the public that sanatoria are not dangerous.

### GRANTS FOR THE MEDICAL TREATMENT OF SCHOOL CHILDREN.

The Board of Education has just issued the Regulations governing the grants which will be made in aid of the provision of medical treatment for children attending public elementary schools. From a supplementary circular local authorities are able to form some idea of the points to which the Board will pay special regard when deciding on the details of the grants. In a general sense the work in respect of which these grants will be made may be described as medical treatment; this will, however, include preparatory measures and the following up of defective children discovered at the school inspections. Before the Board is satisfied as to any measures that have been taken in this direction it will require evidence that the work of inspection itself is being systematically and consistently carried out on the school premises and in school hours. One visit a year will not be regarded as sufficient. It is interesting to note that official approval is now given to an examination rate of eight or ten children an hour. In the early days of school inspection there was much difference of opinion on this point. In assessing grants the Board will take into consideration auxiliary undertakings, if carried out by the school medical officer or his staff, such as re-examinations, refractions, treatment of special cases at clinics and after-care arrangements.

### CO-ORDINATION OF SCHOOL MEDICAL SERVICE AND ATTENDANCE.

It is expected of local authorities that they shall make the fullest possible use of voluntary or other agencies already in existence, and that complete co-ordination shall be secured between the school medical service and the school attendance department, in order that contagious diseases may be fully controlled. The circular referred to lays stress on the importance of including in annual reports a detailed statement of the work done by the medical officers and the school nurses, and it would appear that the preparation of this information will mean a very considerable amount of clerical work and office collaboration if the several authorities are to get their respective grants. The Board of Education is undoubtedly taking steps in the right direction by conferring further powers on local education authorities and providing adequately safeguarded stimulation. With the best intentions in the world of doing nothing to lessen the responsibility of parents or to deter them from seeking the advice of their own medical attendants, there will always remain a large amount of unremedied disease or defect, which will provide a considerable field for the work of public health bodies. And this work will be of great and lasting value, not only in alleviating the sufferings of the children and increasing their capacity to profit by the education provided, but also in laying a foundation of health on which must depend the future physical welfare of the adult population.

### THE STOCKPORT INFIRMARY EXTENSION.

The King Edward VII. Commemoration Fund at Stockport has been now closed, and it is expected that some £7,275 will be handed over to the treasurer of the Infirmary. It has been decided that the Infirmary Committee shall begin at once on the extensions mentioned in the appeal, and Mr. Sydney Hollins states that as regards the Nurses' Home, in two or three weeks the work will be busily proceeding. The funds are expected to be sufficient for all the contemplated alterations. These, it should be remembered, comprised the increase of the accommodation from 88 to 110 beds, and an enlarged casualty and out-patient department together with various improvements in the dispensary and kichen. It is noteworthy that the subscribers to the appeal fund are urging the building to be undertaken without any further delay, with the result that the Building Committee is being approached so that the plans will be decided upon at an early date.

### THE GOVERNMENT, VOLUNTARY AID, AND CONSUMPTION.

Another example of the risk of overlapping in the matter of providing sanatoria comes from Staffordshire. Dr. Reid, the County Medical Officer, has prepared a scheme in which he proposes a central sanatorium for the county with various special hospitals in relation to it, the business of which shall be to provide preliminary treatment. In addition to the central sanatorium and to the special hospitals, he urges the importance of establishing eight dispensaries, four of which should serve large sections of the population, while the remaining four should be travelling dispensaries dealing with outlying and rural districts. If the Local Government Board approve Dr. Reid's scheme it is hoped that half of its large cost, if not more, will be provided by the Exchequer. The basis of the scheme, it will be observed, is the co-operation of the Borough Councils with the County Council. The scheme itself cannot be criticised until it has materialised to some extent, and the attempt to secure co-operation will be welcomed, for co-operation alone can prevent waste and overlapping. But this co-operation to achieve its aim in this respect must be carried still further. For instance, the County Memorial to King Edward is understood to be connected with the prevention of consumption. The Committee in charge of the Memorial must, therefore, make its position and proposals clear and, in short, co-operate also with any county proposition which may be decided on. The danger is the simultaneous establishment of voluntary and State efforts on a large scale to provide sanatoria.

### HOSPITALS AND MURAL DECORATION.

The reaction against pre-Listerian uncleanliness in treatment and absence of hygiene in buildings and rooms for the sick led to a strong prejudice against any unnecessary decoration of hospital buildings and wards. Just as nineteenth-century Gothic was abandoned in favour of the barest simplicity for the outside of hospital buildings, so pictures, carpets, cornices, and wainscots were abandoned for indoor construction, on the obvious principle that all dust collectors were out of place. There is beginning, however, to be a reaction against this stern simplicity, and the problem it is trying to solve is the provision of a style of decora tion that shall be no less hygienic than the undecorated walls. For instance, tile-pictures are gradually becoming more common in children's wards. Another example of the attempt to revive decoration is about to be seen at the Middlesex, now that Mr. Edmund Davis, a governor, has arranged to rebuild the entrance with a view to mural decoration. In this connection the committee of the forthcoming Exhibition of Mural Paintings announce a competition for the decoration of the vestibule of the Middlesex Hospital. The plans will provide for four wall-spaces lit from the roof, each measuring 5 ft. 8 in. high by 10 ft. 11 in. broad. Symbolical subjects are suggested; each competitor is limited to one design, and Mr. Davis offers £100 for the execution of each painting from a design approved by him. If the Middlesex Hospital does not approve the accepted design, £50 will be given in lieu of the execution of the work.

### CHESTERFIELD HOSPITAL AND WORKING-MEN.

During the strike this hospital, which is largely dependent upon the working-men's support, received very little in the way of subscriptions from workmen. But before the strike ended the Pilsley Colliery Company's workmen wrote to Mr. W. Thraves, the Secretary, stating that they were withdrawing from their funds in the bank (£27) their quarter's gift in order not to be behind with their promised help. A day or two following the money was paid over. Such evidences of workingmen's support is a great encouragement to hospitals. Leicester, for instance, provides a splendid example of the value of such co-operation through its Hospital Saturday Society. At Chesterfield it was specially appreciated, as some eighty beds have been occupied throughout the strike period. We hope that such an example of loyalty to pledges will be general.

### THE KING EDWARD MEMORIAL AT LINCOLN.

A rather curious situation has arisen at Lincoln County Hospital from the recommendation of the weekly board that a sum not exceeding £1,100 be spent on the improvement of the casual block. It should be remembered that a scheme embodying this was included in the proposed King Edward Memorial, but, according to Canon H. W. Hutton, the chairman of the quarterly meeting, that is hung up at present. The representations of the medical staff and the obvious need for improvement have, he said, made the scheme absolutely necessary. With this object in view the meeting sanctioned the expenditure, which the secretary-superintendent, Mr. A. More, explained need not come out of the capital account, as there was a sum of £750 in the bank, and a legacy of £1,000 was coming in. The Trades' and Labour Council have drawn attention to the unsatisfactory condition of the out-patient department. The upshot is that an improved casualty block is thought to be so necessary that it cannot afford to wait for the slow progress of the King Edward Memorial Fund. While sympathising with this recognition of urgency on the part of the hospital, the Memorial Fund would appear to be placed in a rather unenviable light, and now that the scheme for which that Fund was intended to provide is to be financed from other sources, to what end will the Memorial Fund be diverted? Is it to contribute its mite to the adopted scheme, or is it to be regarded as a failure?

### THIS WEEK'S DRUG MARKET.

The most important event of the week has been an advance in the price of quinine sulphate of a penny per ounce. For a long time, one might almost write for years, the market for quinine has been in a depressed condition, and the improved tone comes as a relief to holders of the alkaloidal salt, of whom there are many. It is thought by some that the present advance is the precursor of others, but of that it is not possible to speak with authority. The main reason for the higher price is the fact that the shipments of cinchona bark from Java to Europe have of late been noticeably smaller, and as a consequence the price realised for the bark at the Amsterdam sales has been higher. It may be that the shipments of bark will be increased again, and the reason for the lessened shipments is not altogether clear; there are rumours that shippers in Java have come to an understanding to the effect that some rule regulating the quantity shipped shall be observed, but there seems to be no solid foundation for the report. In any case, it would not be altogether unwise to buy quinine at the present time, for notwithstanding the advance in price the article is still comparatively cheap. The price of iron and quinine citrate has also been advanced. Buchu leaves are a trifle dearer. The price of cod-liver oil now appears to have reached something like its lowest level, and it would not be greatly to the advantage of buyers to refrain much longer from making their purchases. Santonin has again been advanced in value.

### OUR SPRING NUMBER.

Our Spring Number will be published next week, April 27. It will contain a series of special articles devoted to every aspect of the Modern Institution Kitchen, many of which will be illustrated. The kitchen in various special and general hospitals will be considered, and there will be an article on the "Continental hospital kitchen," together with an account of many of the most recent appliances for the kitchen and allied departments. These articles will be in addition to the customary features, and the price of the much-enlarged paper will be, as usual, one penny.

